# Renal Papillary Necrosis Caused by Protein C Deficiency Leading to Recurrent Hydronephrosis

**DOI:** 10.1089/cren.2016.0018

**Published:** 2016-02-01

**Authors:** Rohit Kumar Chugh, Vincent Olorunnisomo, Evan James Fowle, Ippolito Modica, Ira Meisels, Mantu Gupta

**Affiliations:** ^1^ Department of Urology, Mount Sinai Health System, New York, New York.; ^2^ Department of Pathology, Mount Sinai Health System, New York, New York.; ^3^ Department of Nephrology, Mount Sinai Health System, New York, New York.

## Abstract

A patient with history of a solitary functioning kidney and protein C deficiency (PCD) presented with recurrent severe hydronephrosis causing acute kidney injury upon chronic kidney disease. Work-up with endoscopic evaluation revealed renal papillary necrosis (RPN) and sloughed renal papillae to be the true cause of the recurrent obstruction. Pathologic evaluation of the sloughed tissue confirmed the diagnosis of RPN. This is the first case reported in the literature illustrating the unique presentation of RPN in the setting of PCD.

## Case Report

**FIG. 1.**
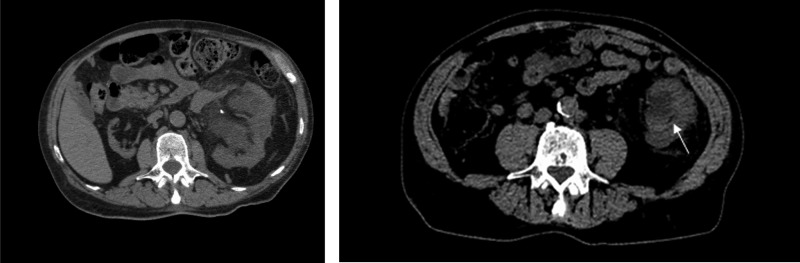
Noncontrast CT scan images. *Left panel* showing severe hydronephrosis of left kidney with a vascular calcification and an atrophic right kidney. *Right panel* showing necrotic debris in renal collecting system (*arrow*).

A 72-year-old male with a past medical history of hypertension, chronic kidney disease, a solitary functioning left kidney (right kidney atrophic from unknown cause), and protein C deficiency (PCD) with baseline creatinine of 4 mg/dL presented with flank pain, and upon evaluation he was found to have severe left hydroureteronephrosis (Fig. 1) with an elevation of his creatinine to 12.2 mg/dL. He underwent a left ureteral stent placement. During the procedure, a small stone in the bladder was identified, presumably passed from the kidney, and it was removed. The stone was sent for analysis and its constituents were unable to be identified, even after repeat analysis.

His renal function and hydronephrosis improved and the stent was removed 9 weeks later, assuming the issue of obstruction was resolved. Two months later, the patient presented with hematuria and flank pain and laboratory evaluation again revealed worsened renal function. A CT scan showed recurrent severe left hydroureteronephrosis with no definite cause of ureteral obstruction. Upon placement of the ureteral stent, a large efflux of bloody urine was noted from the stent.

**FIG. 2.**
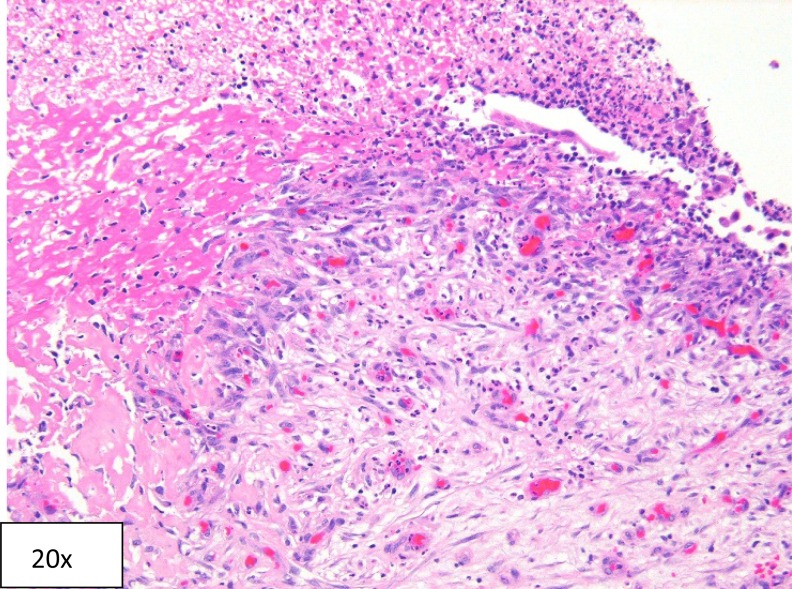
Hematoxylin and eosin section showing necrotic tissue from renal papilla, granulation tissue with young fibroblasts, and abundant vessels with reactive endothelium, and adjacent fibrin with many neutrophils and nuclear debris.

After his renal function returned to baseline, a diagnostic ureteroscopy was performed to assess for presence of ureteral stricture, mass, or an obstructing lesion as well as to remove any nonobstructing calculi within the collecting system. Ureteroscopy and pyeloscopy revealed a significant amount of debris and clot within the collecting system. Papillary necrosis was noted throughout the collecting system, especially notable in the central and lower calices. Floating necrotic renal papillary tissue was also observed in the lower pole of the kidney. There were no stones identified, only small blood clots present in the areas of missing papillae where there was a crater in the place where a papilla was expected. The kidney was irrigated free of all clots and debris. The necrotic material was removed with biopsy forceps to be sent for pathology analysis. Pathologic evaluation was consistent with necrotic renal papillae without any signs of invasion with cancer or infection (Fig. 2). The patient's protein C activity was measured to be 44 IU/dL, which is below the normal reference range (75–133 IU/dL) and considered to be mildly deficient. A ureteral stent was placed at the time of ureteropyeloscopy and removed 2 weeks later. At 6-month follow-up no recurrence was noted.

To our knowledge, this is the first case reported in the literature illustrating a unique presentation of renal papillary necrosis (RPN) in the setting of PCD and in the absence of a locally invasive infection or malignancy.

## Discussion

RPN is characterized by coagulative necrosis of the renal medullary pyramids and papillae brought on by several associated conditions and toxins that exhibit synergism toward the development of ischemia. Owing to the peculiar arrangement of blood supply of renal medulla and papilla, these structures are particularly vulnerable to ischemic necrosis. The blood supply to the medulla and papilla tapers as it approaches the papillary tip, thus resulting in more pronounced coagulative necrosis because of ischemia at the papilla than at the base of pyramid. RPN may be characterized by periods where the patient is completely asymptomatic, but usually presents with gross hematuria with or without flank pain, often mimicking renal colic typically associated with obstructing nephrolithiasis.^1^ It is more likely to be seen in patients with sickle cell trait,^2,3^ analgesic nephropathy,^4^ invasive infections (specifically fungal),^5–7^ and as a chemotherapy-related complication.^8^

Protein C is a vitamin K-dependent protein that is produced in the liver, and once it gets converted to activated protein C (ProC), along with protein S, it provides an anticoagulative effect by inactivating coagulation factors Va and VIIIa. Therefore, protein C plays an active role in the coagulation cascade. The gene for protein C is located on the long arm of chromosome 2 and multiple pathogenic mutations have been reported in the literature. Deficiency of protein C can be quantitative (Type I) or functional (Type II) based on genetic mutations.^9,10^ Immunologic assays help to differentiate between the two subtypes.

PCD is a rare genetic abnormality, which predisposes to thrombophilia and leads to thrombosis, often at unusual sites. RPN in patients with PCD is rare and to our knowledge has not been reported in the literature. The prevalence of PCD is 0.2% to 0.4% in a healthy population, 3.7% in patients in whom deep vein thrombosis was diagnosed, and 4.8% in patients having thrombophilia.^11^

Based on our experience with this case, we recommend having a high index of suspicion for RPN in patients with PCD who present with signs and symptoms of upper urinary tract obstruction.

## Abbreviations Used

CT: computed tomography
PCD: protein C deficiency
RPN: renal papillary necrosis
